# Supplementary material for: The genome sequence of a tachinid fly, Thelaira solivaga (Harris, 1780)
Source: Wellcome Open Res. 2023 Dec 5;8:564. [Version 1] doi: 10.12688/wellcomeopenres.19639.1 (PMC11101925; doi:10.12688/wellcomeopenres.19639.1)
Supplement: Peer review history

# Reviewer report: Reviewer response for version 1

**DOI:** 10.21956/wellcomeopenres.21755.r74609

**Published:** 2024-05-17

**Authors:** Darren J Obbard

**Affiliations:** 1 The University of Edinburgh, Edinburgh, Scotland, UK

This data note reports the sequencing and assembly of the genome of *Thelaira solivaga* as part of the “Darwin Tree of Life” programme. In common with other data notes from this research effort, the reporting is standardized and quite brief. As such, I have very few comments to make.

 The approach is state-of-the-art, the raw data appear to be of a suitably high quality, and the assembly methods are appropriate. The public availability of raw data and genome assembly are appropriate. The resulting genome is likely to be of very high quality, and I have no doubt that it will be of great value to any researchers working on tachinids and other diptera, or on the comparative or evolutionary genomics of insects more generally.

 My minor suggestions are:

 (1) In addition to the sequenced specimen itself, it would be nice to have an ‘in life’ photograph, ideally of both sexes, given the described morphological differences. CC-BY images do seem to be available from Wikimedia Commons and iNaturalist (but may be misidentified *Thelaria nigrina*?).  

 (2) I think a reference (or two) is required for the statement “ *Thelaira solivaga* is recorded from across southern and central Britain …. The species is probably double brooded, with adults on the wing from late April or early May until mid-September.” Or, a “Pers Obs” to make clear that information is not otherwise available in the literature.

 (3) I am surprised that no statements are made regarding the following: (a) Global distribution and abundance, (b) conservation status, (c) value or utility of the sequences provided. I think there maybe a boilerplate paragraph missing from the end of the background? It usually reads: “Here we present a chromosomally complete genome sequence for …. This project is a collaborative effort to sequence all named eukaryotic species in the Atlantic Archipelago of Britain and Ireland.”

 (4) I notice that RNAseq data have also been generated from the abdomen of the same specimen (ERR11641108), and I therefore think this should be mentioned in the paper.

Are sufficient details of methods and materials provided to allow replication by others?

Yes

Is the rationale for creating the dataset(s) clearly described?

No

Are the datasets clearly presented in a useable and accessible format?

Yes

Are the protocols appropriate and is the work technically sound?

Yes

Reviewer Expertise:

Evolutionary genetics and genomics of invertebrates (Drosophila) and their pathogens (viruses).

I confirm that I have read this submission and believe that I have an appropriate level of expertise to confirm that it is of an acceptable scientific standard.

# Reviewer report: Reviewer response for version 1

**DOI:** 10.21956/wellcomeopenres.21755.r81677

**Published:** 2024-05-15

**Authors:** Josie Paris

**Affiliations:** 1 Department of Life and Environmental Science (Di.S.V.A.), Marche Polytechnic University, Ancona, Ancona, Italy; 2 Department of Health, University of L'Aquila, Coppito, Italy

This report provides details on the assembly of the tachinid fly Thelaria solivaga, found in southern and central Britain. The larvae of the species are parasitic of Tiger moths. The genome has been assembled and annotated as part of the Darwin Tree of Life Project. The genome (429.3 Mb, scaffold N50 = 77.2 Mb) was assembled using the latest sequencing technologies (PacBio Hifi data, scaffolded with Hi-C chromosome conformation data). After manual curation of several missing joins or mis-joins, the scaffolded assembly is of high quality, with a good QV score and 100% kmer completeness, and a BUSCO completeness of 99%. No annotation is reported. The genome and its data are found on the ENA.

 Major comments:

 1.The background on the species is rather brief. Perhaps not much is known about this species, but it would be nice to see more information on the species (for example its lifespan?) and its distribution (perhaps also outside of Britain?) included. 

 2. No annotation has been performed on the genome. If due to difficulties in obtaining RNAseq data, I suggest either a preliminary annotation is performed using existing protein data of related species, or else the authors should include some reasoning as to why there is no annotation

 Minor comments:

 1. Sufficient details are provided in the Methods, except there is no mention of the amount of Hi-C data generated (and its coverage)

 2. For the mitochondrial assembly using MitoHiFi, it's not clear which algorithm was used - MitoFinder or MITOS?

Are sufficient details of methods and materials provided to allow replication by others?

Partly

Is the rationale for creating the dataset(s) clearly described?

Partly

Are the datasets clearly presented in a useable and accessible format?

Yes

Are the protocols appropriate and is the work technically sound?

Yes

Reviewer Expertise:

population genomics, transcriptomics, genome assembly and annotation of non-model species.

I confirm that I have read this submission and believe that I have an appropriate level of expertise to confirm that it is of an acceptable scientific standard, however I have significant reservations, as outlined above.

# Reviewer report: Reviewer response for version 1

**DOI:** 10.21956/wellcomeopenres.21755.r81667

**Published:** 2024-05-08

**Authors:** Ravikumar Dodiya

**Affiliations:** 1 Sardarkrushinagar Dantiwada Agricultural University, Sardarkrushinagar, Dantiwada, India

The study presents a comprehensive genome assembly of an individual male *Thelaira solivaga*, a tachinid fly belonging to the order Diptera, with a genome sequence spanning 429.3 megabases. The assembly is scaffolded into 7 chromosomal pseudomolecules, including the X and Y sex chromosomes, and the mitochondrial genome is also assembled to a length of 21.09 kilobases. *Thelaira solivaga* is described as a medium-sized tachinid fly, with distinct characteristics in males and females. The larvae of this species are internal parasites of various species of Tiger Moths (Lepidoptera: Erebidae), with eggs laid externally onto the host and multiple larvae developing within a single host. The species is found across southern and central Britain, possibly double-brooded, with adults observed from late April or early May until mid-September. The genome was sequenced from a male specimen collected from Wytham Woods, Oxfordshire, UK, with sequencing data generated using Pacific Biosciences HiFi circular consensus DNA sequencing and Hi-C data. The assembly process involved scaffolding with Hi-C data and manual curation to correct assembly errors and remove haplotypic duplications. The final assembly comprises 26 sequence scaffolds, with 99.86% assigned to 7 chromosomal-level scaffolds, including autosomes and sex chromosomes. Quality assessment indicates a high-quality assembly with a QV of 63.8, 100% k-mer completeness, and 99.0% BUSCO completeness using the diptera_odb10 reference set. Software tools such as Hifiasm, purge_dups, YaHS, and Merqury were used for assembly and evaluation, and the genome was analyzed within the BlobToolKit environment. The study provides valuable insights into the genomics of *Thelaira solivaga*, which could contribute to further research on its biology, ecology, and interactions with host species.

**In Background informstion:**

 Write super parasitism or multiple parasitism behavior seen in *Thelaira solivaga* instead of this  Eggs are laid externally directly onto the host, with multiple larvae developing within a single host. 

 Provide the exact latitude and longitude of location w from where the sample collected.

 WHY ONLY TAKEN MALE of *Thelaira solivaga *?

 is there a lack of availability of sample?

Are sufficient details of methods and materials provided to allow replication by others?

Yes

Is the rationale for creating the dataset(s) clearly described?

Yes

Are the datasets clearly presented in a useable and accessible format?

Yes

Are the protocols appropriate and is the work technically sound?

Yes

Reviewer Expertise:

Biological control

I confirm that I have read this submission and believe that I have an appropriate level of expertise to confirm that it is of an acceptable scientific standard, however I have significant reservations, as outlined above.
